# Supplementary material for: Rbm20ΔRRM Mice, Expressing a Titin Isoform with Lower Stiffness, Are Protected from Mechanical Ventilation-Induced Diaphragm Weakness
Source: Int J Mol Sci. 2022 Dec 10;23(24):15689. doi: 10.3390/ijms232415689 (PMC9779751; doi:10.3390/ijms232415689)
Supplement: Supplementary file 1 [file ijms-23-15689-s001.zip › ijms-2058194-supplementary.pdf]

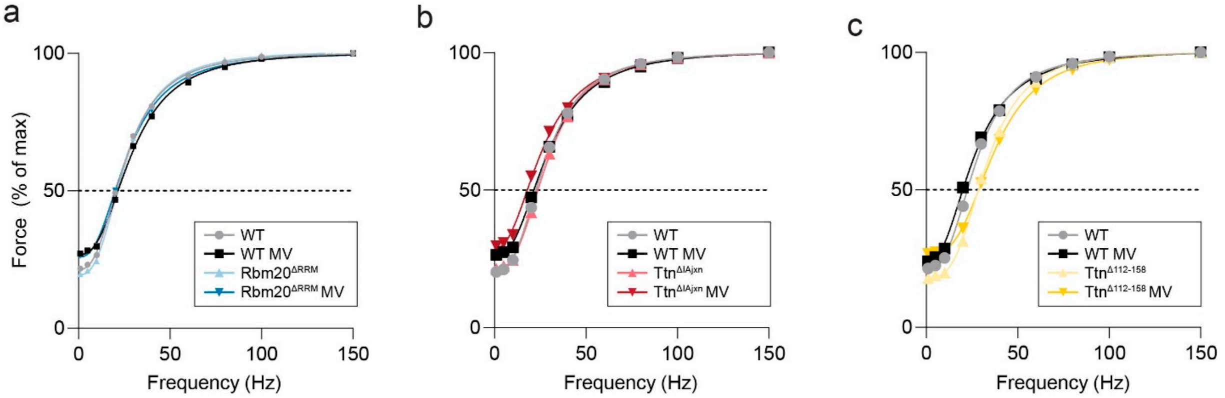

Figure S1. Absolute Force-Frequency relations.
(a,b,c) Fitted curves for the averaged tension-stimulation frequency relation, relative to maximal tension in in Rbm20$^{\Delta RRM}$, Ttn$^{\Delta IAjxn}$ and Ttn$^{\Delta 112\text{-}158}$ respectively. F50 was determined as the absolute EC50 of the curve, indicated by the dotted line, and therefore represents the frequency required to develop 50% of maximal force. WT = wild type, MV = mechanically ventilated. Each dot represents a single animal and animals are only presented in each graph once.

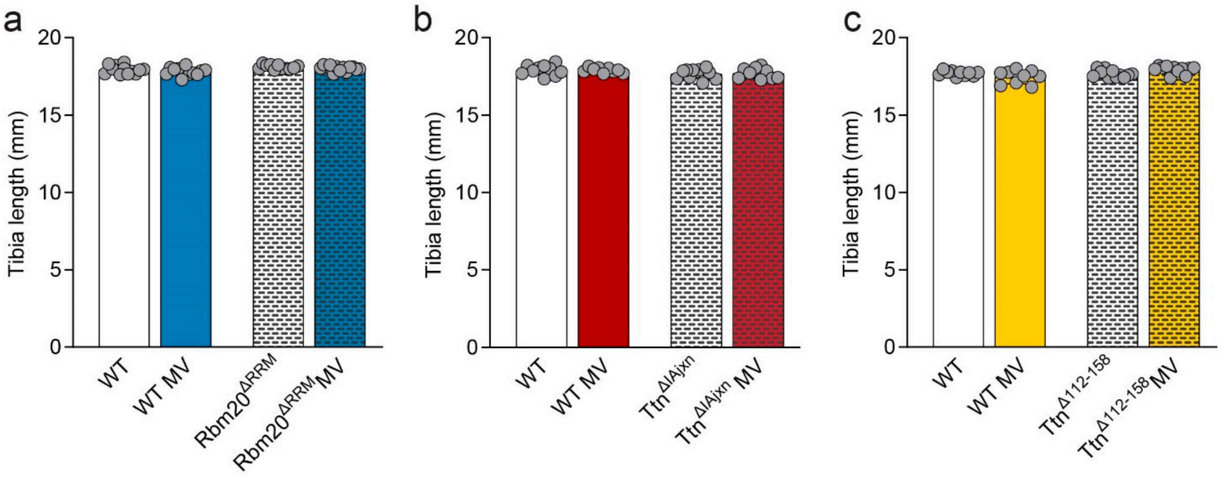

Figure S2. Tibia length
(a,b,c) Tibia length was comparable between non-ventilated and ventilated mice, and between WT and Rbm20$^{\Delta RRM}$, Ttn$^{\Delta IAjxn}$ and Ttn$^{\Delta 112\text{-}158}$ mice respectively. Each dot represents a single animal and animals are only presented in each graph once.

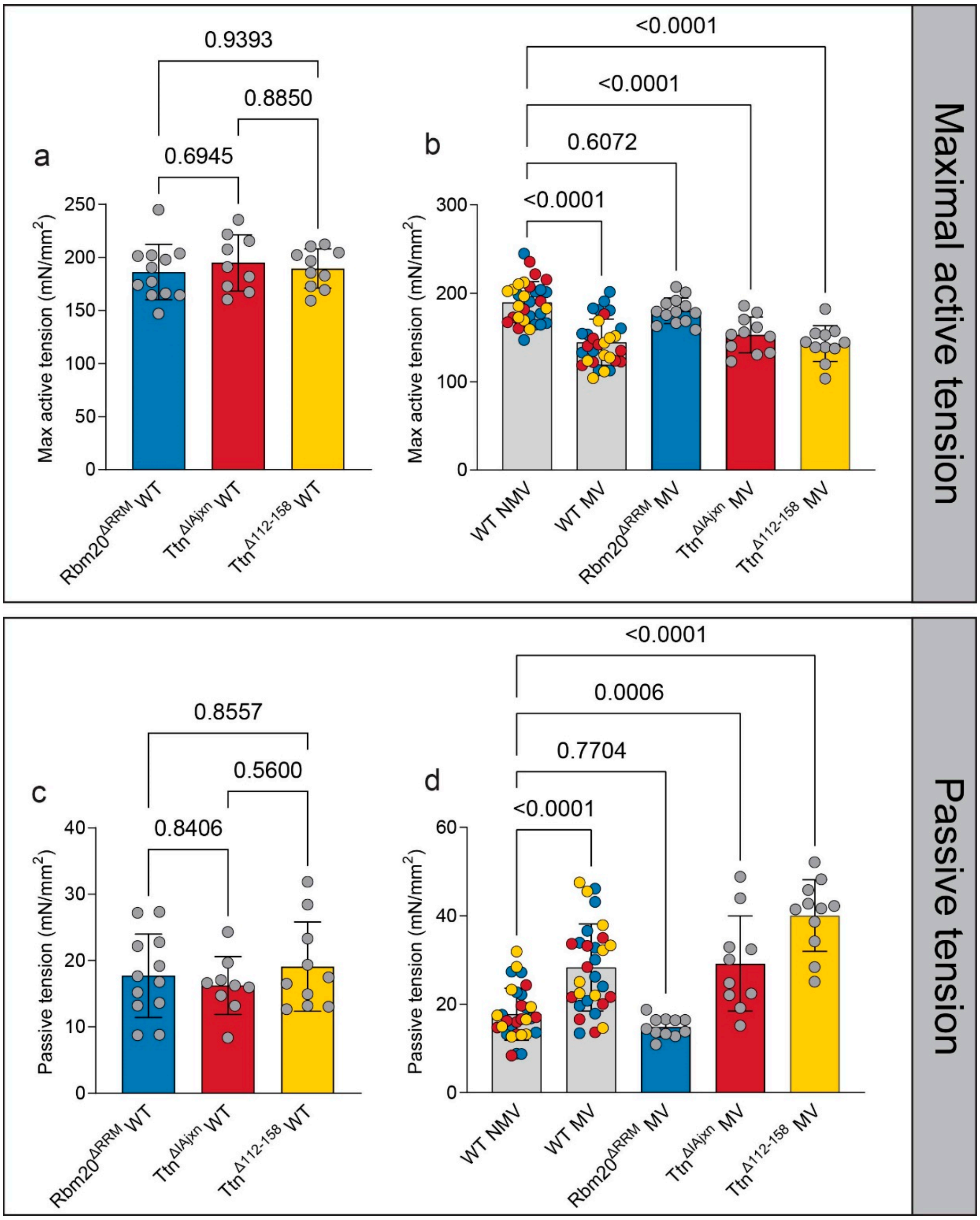

Figure S3. Model comparisons

Littermate wild-type mice were used for each of the three models. (a) Maximal active tension is similar across the non-mechanically ventilated wild-type mice from different models. (b) The maximal active tension is reduced in the mechanically ventilated wild-type-, $Ttn^{\Delta IAjxn}$-, and $Ttn^{\Delta 112\text{-}158}$ mice, but not in the mechanically ventilated $Rbm20^{\Delta RRM}$ mice, compared to the non-mechanically ventilated wild-type mice pooled from all three models. (c) Passive tension is similar across the non-mechanically ventilated wild-type mice from the different models. (d) The passive tension is increased in the mechanically ventilated wild-type-, $Ttn^{\Delta IAjxn}$-, and $Ttn^{\Delta 112\text{-}158}$ mice, but not in the mechanically ventilated $Rbm20^{\Delta RRM}$ mice, compared to the non-mechanically ventilated wild-type mice pooled from all three models. Bars represent mean ± SD. * $p<0.05$, ** $p<0.01$, *** $p<0.001$, **** $p<0.0001$. WT = wild type, MV = mechanically ventilated. Each dot represents a single animal and animals are only presented in each graph once.
